# Risk and Protective Factors for Vaping and Smoking Among High School Students in Maine

**DOI:** 10.5888/pcd20.220307

**Published:** 2023-04-20

**Authors:** Gabriel Tilton, Sara Huston, Pamela Albert

**Affiliations:** 1 University of Southern Maine, Portland, Maine; 2 Maine Center for Disease Control and Prevention, Augusta, Maine

## Abstract

**Introduction:**

Vaping rates are rising among adolescents across the country, and smoking rates remain high. Understanding risk and protective factors associated with vaping and smoking can guide public health interventions. This study examined risk and protective factors associated with vaping and smoking among high school students in Maine.

**Methods:**

We used 2019 Maine Integrated Youth Health Survey (MIYHS) data to examine risk and protective factors for vaping and smoking among Maine high school students. Our analytic sample consisted of 17,651 Maine high school students. In addition to bivariate analyses, we used unadjusted and adjusted logistic regression models to assess risk and protective factors.

**Results:**

Factors with the greatest effect on students’ likelihood to vape, smoke, or do both were parental attitude toward adolescent smoking and depressive symptoms. Students who reported their parents feel it is a little wrong or not wrong at all if they smoked had 4.9 times higher adjusted odds of smoking and 4.6 times higher adjusted odds of vaping and smoking compared with students who said their parents feel it would be wrong or very wrong if they smoked. Students who reported depressive symptoms had 2.1 times higher adjusted odds of vaping, 2.7 times higher adjusted odds of smoking, and 3.0 times higher adjusted odds of vaping and smoking compared with students who did not report depressive symptoms.

**Conclusion:**

Understanding risk and protective factors for smoking and vaping among high school students can help tailor adolescent-focused vaping and smoking public health interventions to increase effectiveness.

**Summary**

**What is already known on this topic?**

Vaping rates among high school students have increased in recent years, while smoking rates have gradually decreased but remain high.

**What is added by this report?**

This study revealed several factors that increase the odds of vaping, smoking, or both among high school students in Maine, including depressive symptoms and how students perceive a parent’s or a guardian’s attitude toward adolescent smoking.

**What are the implications for public health practice?**

The information gained from this study can be used to tailor public health interventions that are focused on adolescents at high risk of vaping and smoking.

## Introduction

As of 2019, the smoking rate among high school students nationally in the US was 5.8%, and the e-cigarette/vaping rate was 27.5%; local rates were 7.1%, and 28.7%, respectively, in Maine (1–3). Recent research has shed light on factors that increase the risk of smoking and vaping among adolescents. For example, adolescents who use e-cigarettes were found to be at least 3 times more likely than those who did not use e-cigarettes to start smoking combustible cigarettes (4–8). Adolescents who had friends who smoked or used e-cigarettes, or friends who were accepting of cigarette use, were more likely than those who did not have such friends to initiate use of e-cigarettes (6,8). In addition, depressive symptoms, living with someone who uses e-cigarettes or cigarettes, and poor school performance increase an adolescent’s likelihood to start smoking or vaping (9–11). Several protective factors may decrease an adolescent’s risk of initiating e-cigarette use, such as the belief that using an e-cigarette could lead to addiction, parental antismoking norms, college aspirations, and higher parental educational attainment (4,12,13). Research gaps exist on the risk and protective factors for vaping and smoking among adolescents, including research on the effect of these factors among Maine high school students. The objective of this study was to explore risk and protective factors associated with vaping and smoking among high school students in Maine.

## Methods

We used data on high school students from the 2019 Maine Integrated Youth Health Survey (MIYHS), a large biennial cross-sectional survey of students in grades 5 through 12 in Maine. More information on the MIYHS is available at www.maine.gov/miyhs. All public and quasi-public high schools in the state are invited to participate in the survey. MIYHS data are self-reported by students through paper-based surveys completed in classrooms. The survey has questions on demographic characteristics and health-related behaviors and attitudes. The MIYHS high school survey has 4 versions: A, B, C, and D. Participating high school classes are randomly assigned a version, and version C is Maine’s Youth Risk Behavior Survey. Some questions (“core”) appear on all 4 survey versions, while other questions (“noncore”) appear on 1 or 2 versions. Although the question on smoking is a core question, the question on vaping was not in 2019. Our analytic sample consisted of 17,651 Maine high school students who completed survey version C or D, the 2 versions with questions on vaping. The overall response rate was 60.5% for version C, 63.1% for version D, and 61.9% for the combined versions. Schools mailed and emailed an informational letter to all parents for passive consent. Students were not surveyed if their parent declined their participation or if the letter was returned as undeliverable. The MIYHS was approved by the University of Southern Maine Institutional Review Board, and the research presented here was deemed exempt by that review board.

### Variables

Students who vaped or smoked in the past 30 days were categorized into 3 groups: those who smoked in the past 30 days (answering 1 or more days to “During the past 30 days, on how many days did you smoke cigarettes?”), those who vaped in the past 30 days (answering 1 or more days to “During the past 30 days, on how many days did you use an electronic vapor product?”), and those who smoked and vaped in the past 30 days, to provide our outcome variables of interest. These 3 outcome variables were not mutually exclusive.

Demographic variables used in analyses were sex, sexual orientation, race, ethnicity, and grade level. We used the county of school location to examine and adjust for geographic differences across the state.

We assessed the following risk and protective factors: students’ perception of smoking as a health risk (“How much do you think people risk harming themselves [physically or in other ways] if they smoke one or more packs of cigarettes per day?”), parental attitude toward their adolescent’s smoking (“How wrong do your parents feel it would be for you to smoke cigarettes?”), place of sleep most nights (as an indicator of housing instability, “During the past 30 days, where did you usually sleep?”), depressive symptoms during the past year (“During the past 12 months, did you ever feel so sad or hopeless almost every day for two weeks or more in a row that you stopped doing some usual activities?”), bullied during the past year (“During the past 12 months, have you ever been bullied on school property?” and “During the past 12 months, have you ever been electronically bullied? Count being bullied through texting, Instagram, Facebook, or other social media.”), physical activity level (“During the past 7 days, on how many days were you physically active for a total of at least 60 minutes per day? Add up all the time you spent in any kind of physical activity that increased your heart rate and made you breathe hard some of the time.”), average grades in school (“During the past 12 months, how would you describe your grades in school?”), talking to parents about school (“How often does one of your parents talk with you about what you are doing in school?”), feeling like a teacher cares (“Do you agree or disagree that at least one of your teachers really cares and gives you help and support when you need it?”), and family love and support (“How often does your family give you love and support?”). MIYHS questionnaires can be found at www.maine.gov/miyhs/2019-survey-information.

### Data analyses

We used SAS Enterprise Guide 5.1 (SAS Institute, Inc) to perform all analyses and SAS Survey Procs to account for complex survey design. Data were weighted to adjust for nonresponse and to be representative of the Maine high school population; all percentages were weighted. We used χ^2^ tests to conduct bivariate analyses of demographic variables, county, risk and protective variables, and outcomes of interest. We performed multiple logistic regression analyses to obtain odds ratios (ORs), 95% CIs, and *P* values for risk and protective factors first while adjusting for county and demographic characteristics and then while adjusting for county, demographic, and all risk and protective factors. Regression models included students with no missing data on all variables in that model. Significance was set at *P* ≤.05. The final models were built through an elimination process. All variables were included in a full model; any variables with a *P* >.10 were removed and examined to ensure that removal did not affect other ORs. If removal did not affect other ORs, the variable was eliminated from the final model.

## Results

Approximately 28.7% of Maine high school students reported vaping in the past 30 days, 6.6% reported smoking in the past 30 days, and 5.3% reported vaping and smoking in the past 30 days (Table 1). These findings suggest that most students who smoke also vape, but most students who vape do not also smoke. 

**Table 1:** Demographic, County, and Risk and Protective Factors Among Maine High School Students Who Reported Vaping, Smoking, and Smoking and Vaping in the Past 30 Days, 2019 Maine Integrated Youth Health Survey

Characteristic	Vaped in the past 30 days (n = 16,692)			Smoked in the past 30 days (n = 16,980)			Smoked and vaped in the past 30 days (n = 16,337)		
	No.	Weighted % (95% CI)	*P* a	No.	Weighted % (95% CI)	*P* a	No.	Weighted % (95% CI)	*P* a
**Total**	4,703	28.7 (27.4–29.9)	<.001	1,135	6.6 (6.1–7.2)	<.001	862	5.3 (4.8–5.8)	<.001
**Sex**									
Female	2,469	29.8 (28.4–31.2)	.003	489	5.8 (5.1–6.4)	.003	395	4.8 (4.2–5.4)	.07
Male	2,173	27.3 (25.8–28.9)		607	7.2 (6.4–8.0)		443	5.6 (4.9–6.2)	
**Sexual orientation**									
Heterosexual	3,865	28.8 (27.6–30.0)	<.001	771	5.6 (5.0–6.2)	<.001	597	4.6 (4.1–5.1)	<.001
Gay or lesbian	102	24.6 (18.7–30.4)		53	11.6 (8.5–14.7)		35	7.6 (4.8–10.4)	
Bisexual	541	32.1 (29.2–35.1)		196	11.7 (9.9–13.4)		154	9.3 (7.7–10.8)	
Not sure	156	20.5 (16.7–24.3)		86	9.8 (7.5–12.1)		58	7.0 (4.7–9.2)	
**Race**									
American Indian or Alaska Native	146	34.3 (29.4–39.2)	<.001	52	12.0 (9.5–14.5)	<.001	35	8.6 (5.8–11.4)	<.001
Asian	105	19.7 (15.4–24.0)		49	9.3 (6.6–12.0)		26	5.2 (3.0–7.5)	
Black or African American	152	25.3 (19.8–30.8)		45	7.6 (4.6–10.5)		35	6.2 (3.6–8.8)	
Native Hawaiian or Pacific Islander	43	55.5 (42.3–68.7)		18	27.9 (16.7–39.0)		14	21.3 (12.0–30.7)	
White	3,915	28.6 (27.3–29.9)		865	6.2 (5.7–6.8)		675	5.0 (4.5–5.5)	
Multiple races	236	29.7 (26.3–33.0)		72	9.0 (6.9–11.1)		53	6.6 (4.9–8.3)	
**Ethnicity**									
Hispanic or Latino	290	33.4 (29.4–37.4)	.01	104	11.3 (8.7–14.0)	<.001	79	9.3 (6.9–11.7)	<.001
Not Hispanic or Latino	4,331	28.5 (27.3–29.8)		985	6.3 (5.8–6.9)		755	5.1 (4.6–5.5)	
**Grade**									
9 (Freshman)	891	19.2 (17.5–21.0)	<.001	181	3.7 (2.9–4.5)	<.001	123	2.5 (1.9–3.1)	<.001
10 (Sophomore)	1,229	27.0 (25.4–28.7)		282	6.1 (5.3–6.9)		221	5.1 (4.3–5.8)	
11 (Junior)	1,252	32.3 (30.2–34.4)		306	7.3 (6.2–8.3)		233	5.8 (4.8–6.7)	
12 (Senior)	1,243	35.7 (33.6–37.8)		323	8.8 (7.9–9.7)		255	7.3 (6.4–8.2)	
**County**									
Androscoggin	322	28.5 (24.1–32.9)	.99	76	6.2 (4.0–8.3)	<.001	49	4.3 (3.0–5.5)	<.001
Aroostook	160	28.6 (21.2–36.1)		52	10.0 (8.9–11.0)		37	7.4 (5.6–9.1)	
Cumberland	1,231	29.1 (26.7–31.5)		222	5.0 (4.1–5.9)		165	3.9 (3.3–4.6)	
Franklin	153	30.8 (27.5–34.2)		50	9.7 (8.2–11.2)		37	7.1 (4.8–9.4)	
Hancock	152	28.9 (20.5–37.3)		43	8.1 (5.5–10.7)		32	6.2 (3.9–8.5)	
Kennebec	347	30.2 (27.2–33.1)		71	5.9 (3.9–7.9)		62	5.3 (3.4–7.3)	
Knox	129	27.8 (21.3–34.3)		42	8.5 (4.0–13.0)		34	7.1 (3.0–11.3)	
Lincoln	126	27.6 (19.0–36.2)		35	7.5 (4.9–10.1)		29	6.7 (4.5–8.9)	
Oxford	287	31.0 (26.7–35.2)		100	10.1 (7.1–13.2)		74	7.9 (5.1–10.8)	
Penobscot	440	25.8 (20.6–31.0)		124	7.1 (4.9–9.3)		99	5.8 (3.9–7.8)	
Piscataquis	47	29.1 (28.8–29.3)		16	9.5 (9.4–9.7)		11	6.5 (4.6–8.3)	
Sagadahoc	136	28.9 (21.9–35.8)		30	7.3 (3.4–11.2)		23	5.9 (2.8–9.1)	
Somerset	266	30.0 (23.1–36.9)		84	9.5 (6.3–12.6)		67	8.0 (4.9–11.0)	
Waldo	44	25.2 (21.3–29.0)		15	8.6 (5.7–11.5)		11	6.6 (3.5–9.7)	
Washington	100	29.5 (20.9–38.1)		45	11.0 (8.0–14.0)		35	9.1 (6.8–11.4)	
York	763	28.1 (25.4–30.9)		130	4.3 (3.0–5.7)		97	3.3 (2.2–4.3)	
**Students’ view of the risk of smoking on one's health**									
No risk or slight risk	645	36.8 (33.8–39.8)	<.001	292	14.9 (13.1–16.7)	<.001	200	11.2 (9.6–12.9)	<.001
Moderate risk or great risk	4,009	27.7 (26.4–28.9)		800	5.5 (4.9–6.0)		643	4.5 (4.0–5.0)	
**How wrong do your parents feel it would be for you to smoke cigarettes**									
A little wrong or not wrong at all	471	54.7 (51.4–58.1)	<.001	311	35.1 (31.7–38.5)	<.001	237	28.9 (25.6–32.2)	<.001
Wrong or very wrong	4,181	27.2 (25.9–28.4)		774	4.9 (4.4–5.3)		606	4.0 (3.6–4.4)	
**Where do you sleep most nights**									
Parents’ house or school housing	4,400	27.9 (26.6–29.1)	<.001	923	5.7 (5.2–6.2)	<.001	713	4.6 (4.1–5.0)	<.001
Other	293	54.0 (49.0–59.1)		206	35.9 (30.7–41.1)		145	29.0 (24.4–33.7)	
**During the past 12 months did you ever feel sad or hopeless for 2 or more weeks in a row**									
Depressive symptoms	2,199	41.6 (39.6–43.6)	<.001	624	11.6 (10.5–12.7)	<.001	516	10.0 (8.9–11.0)	<.001
No depressive symptoms	2,357	22.3 (21.0–23.6)		396	3.6 (3.1–4.0)		288	2.7 (2.3–3.1)	
**Bullied online or at school in the past 12 months**									
Bullied	1,891	38.1 (36.4–39.8)	<.001	498	9.8 (8.8–10.8)	<.001	405	8.3 (7.5–9.1)	<.001
Not bullied	2,702	24.4 (22.9–25.8)		545	4.7 (4.1–5.3)		413	3.7 (3.2–4.3)	
**In the past 7 days, how many days were you physically active for a total of at least 60 min**									
At least 5 days a week	1,855	27.8 (26.2–29.4)	.24	308	4.5 (3.9–5.1)	<.001	243	3.6 (3.1–4.2)	<.001
Less than 5 days a week	2,508	28.8 (27.3–30.2)		703	7.8 (7.0–8.6)		535	6.2 (5.4–6.9)	
**Grades**									
Mostly A’s and B’s	3,059	25.5 (24.2–26.8)	<.001	557	4.4 (4.0–4.9)	<.001	426	3.5 (3.1–3.9)	<.001
Mostly C’s, D’s, or F’s	1,075	43.2 (41.1–45.4)		352	13.7 (12.1–15.3)		287	11.7 (10.3–13.1)	
**How often does one of your parents talk to you about school**									
About every day or once or twice a week	3,287	26.4 (25.1–27.7)	<.001	644	5.0 (4.5–5.6)	<.001	504	4.1 (3.6–4.6)	<.001
Once or twice a month or less	1,026	37.6 (35.6–39.6)		343	11.9 (10.6–13.2)		263	9.6 (8.4–10.9)	
**Do you agree or disagree that at least one of your teachers really cares and gives you support when you need it**									
Strongly agree or agree	3,258	26.2 (24.9–27.4)	<.001	645	5.0 (4.4–5.6)	<.001	493	4.0 (3.5–4.5)	<.001
Not sure or disagree or strongly disagree	1,071	38.3 (35.9–40.7)		340	11.5 (10.1–13.0)		271	9.6 (8.4–10.9)	
**How often does your family give you love and support**									
Most of the time or all of the time	3,100	25.7 (24.4–27.0)	<.001	528	4.3 (3.8–4.9)	<.001	434	3.7 (3.2–4.1)	<.001
Little or no family love and support	1,162	40.5 (38.0–43.1)		446	14.8 (13.1–16.4)		329	11.6 (10.1–13.1)	

a Determined by χ^2^ tests; *P* < .05 considered significant.

### Demographic characteristics

After adjustment for all demographic characteristics and school county, male students had 12% lower odds of vaping (aOR, 0.9; 95% CI, 0.9–1.0; *P* = .002) than their female counterparts; however, their odds of smoking were 44% higher (aOR, 1.4; 95% CI, 1.2–1.7; *P* < .001) and their odds of smoking and vaping were 32% higher (aOR, 1.3; 95% CI, 1.1–1.6; *P* = .001). Maine high school students who identified as bisexual had greater odds of smoking (aOR, 2.4; 95% CI, 2.0–2.9; *P* < .001) or vaping and smoking (aOR, 2.2; 95% CI, 1.8–2.7; *P* < .001) compared with students who identified as heterosexual. Although students who identified as gay or lesbian had lower odds of vaping (aOR, 0.7; 95% CI, 0.5–1.0; *P* = .02) than students who identified as heterosexual, their odds of smoking were higher (aOR, 2.0; 95% CI, 1.4–2.7; *P* < .001). American Indian and Alaska Native students had higher odds of vaping (aOR, 1.4; 95% CI, 1.1–1.8; *P* = .002), smoking (aOR, 2.2; 95% CI, 1.6–2.9; *P* < .001), and vaping and smoking (aOR, 1.9; 95% CI, 1.2–2.9; *P* = .006) than White students. Asian students had lower odds of vaping (aOR, 0.5; 95% CI, 0.4–0.7; *P* < .001) than White students. Hispanic or Latino students had 63% higher odds of smoking (aOR, 1.6; 95% CI, 1.3–2.1; *P* < .001) and 67% higher odds of vaping and smoking (aOR, 1.7; 95% CI, 1.3–2.2; *P* < .001) than students who were not Hispanic or Latino. The prevalence of vaping, smoking, and vaping and smoking increased with each grade level: the odds of vaping, smoking, or vaping and smoking were 2.4 (aOR, 2.4; 95% CI, 2.1–2.7; *P* < .001), 2.5 (aOR, 2.5; 95% CI, 2.0–3.1; *P* < .001), and 3 times (aOR, 3.0; 95% CI, 2.3–3.9; *P* < .001) higher among seniors, respectively, than among their freshman counterparts. Vaping did not vary significantly by school county. However, smoking, and combined vaping and smoking, tended to be higher in more rural counties, such as Washington (smoking, aOR, 2.6; 95% CI, 1.9–3.7; *P* < .001) (vaping and smoking, aOR, 2.6; 95% CI, 1.8–3.8; *P* < .001) and Aroostook (smoking, aOR, 2.4; 95% CI, 1.9–3.0; *P* < .001) (smoking and vaping aOR, 2.1; 95% CI, 1.5–2.8; *P* < .001), after adjusting for demographic characteristics. 

### Vaping

In bivariate analysis, all risk and protective factors, except physical activity, were significantly associated with vaping in the past 30 days (Table 1), and these associations remained significant after adjusting for demographic characteristics and school county (Table 2).

**Table 2:** Adjusted Odds Ratios for Vaping, Smoking, and Vaping and Smoking by Risk and Protective Factors Among Maine High School Students, 2019 Maine Integrated Youth Health Survey

Factor	Adjusted odds ratio^a^ (95% CI)		
	Vaped in the past 30 days	Smoked in the past 30 days	Vaped and smoked in the past 30 days
**Students view of the risk of smoking on one’s health**			
No risk or slight risk	1.6 (1.4–1.8)	2.8 (2.4–3.4)	2.5 (2.0–3.1)
Moderate risk or great risk	1 [Reference]	1 [Reference]	1 [Reference]
**How wrong do your parents feel it would be for you to smoke cigarettes**			
A little wrong or not wrong at all	3.1 (2.7–3.6)	8.9 (7.5–10.6)	8.4 (6.9–10.1)
Wrong or very wrong	1 [Reference]	1 [Reference]	1 [Reference]
**Where do you sleep most nights**			
Parents house or school housing	1 [Reference]	1 [Reference]	1 [Reference]
Other	2.7 (2.2–3.4)	6.1 (4.8–7.9)	5.8 (4.6–7.4)
**During the past 12 months did you ever feel sad or hopeless for 2 or more weeks in a row**			
Depressive symptoms	2.7 (2.5–2.9)	3.5 (3.0–4.1)	4.0 (3.3–4.8)
No depressive symptoms	1 [Reference]	1 [Reference]	1 [Reference]
**Bullied online or at school in the past 12 months**			
Bullied	2.1 (1.9–2.3)	2.2 (1.9–2.6)	2.4 (2.1–2.7)
Not bullied	1 [Reference]	1 [Reference]	1 [Reference]
**In the past 7 days, how many days were you physically active for a total of at least 60 min**			
At least 5 days a week	1 [Reference]	1 [Reference]	1 [Reference]
Less than 5 days a week	1.0 (0.9–1.1)	1.6 (1.3–1.9)	1.6 (1.3–2.0)
**Grades**			
Mostly A’s and B’s	1 [Reference]	1 [Reference]	1 [Reference]
Mostly C’s, D’s, or F’s	2.4 (2.2–2.6)	3.4 (2.9–4.0)	3.6 (3.1–4.3)
**How often does one of your parents talk to you about school**			
About every day or once or twice a week	1 [Reference]	1 [Reference]	1 [Reference]
Once or twice a month or less	1.7 (1.6–1.9)	2.3 (2.0–2.6)	2.3 (1.9–2.7)
**Do you agree or disagree that at least one of your teachers really cares and gives you support when you need it**			
Strongly agree or agree	1 [Reference]	1 [Reference]	1 [Reference]
Not sure or disagree or strongly disagree	1.9 (1.7–2.1)	2.7 (2.2–3.2)	2.8 (2.3–3.5)
**How often does your family give you love and support**			
Most of the time or all of the time	1 [Reference]	1 [Reference]	1 [Reference]
Little or no family love and support	2.0 (1.8–2.2)	3.3 (2.8–3.8)	3.0 (2.5–3.6)

a Adjusted for demographics (sex, sexual orientation, race, ethnicity, and grade level) and school county.

In the final model (Table 3), ORs were attenuated but remained significant for all risk and protective factors. The factors with the strongest associations with vaping were depressive symptoms in the past year (aOR, 2.1), feeling like their parents think it is a little wrong or not wrong at all for them to smoke (aOR, 2.0), average grades of C or lower (aOR, 1.9), being bullied in the past year (aOR, 1.6), and housing instability (aOR, 1.6). Physical activity was not associated with vaping in bivariate analysis or after adjusting for demographic characteristics and school county, but it was associated with a decreased odds of vaping in the final model, after adjusting for other potential risk and protective factors.

**Table 3:** Final Model — Adjusted Odds Ratios for Vaping, Smoking, and Vaping and Smoking by Risk and Protective Factors Among Maine High School Students, 2019 Maine Integrated Youth Health Survey

Factor	Adjusted odds ratio^a^ (95% CI)		
	Vaped in the past 30 days	Smoked in the past 30 days	Vaped and smoked in the past 30 days
**Students’ view of the risk of smoking on one’s health**			
No risk or slight risk	1.4 (1.2–1.7)	2.1 (1.6–2.6)	2.0 (1.5–2.6)
Moderate risk or great risk	1 [Reference]	1 [Reference]	1 [Reference]
**How wrong do your parents feel it would be for you to smoke cigarettes**			
A little wrong or not wrong at all	2.0 (1.7–2.4)	4.9 (3.8–6.4)	4.6 (3.5–6.0)
Wrong or very wrong	1 [Reference]	1 [Reference]	1 [Reference]
**Where do you sleep most nights**			
Parents’ house or school housing	1 [Reference]	1 [Reference]	1 [Reference]
Other	1.6 (1.2–2.1)	2.4 (1.7–3.4)	2.6 (1.9–3.6)
**During the past 12 months did you ever feel sad or hopeless for 2 or more weeks in a row**			
Depressive symptoms	2.1 (1.9–2.3)	2.7 (2.2–3.4)	3.0 (2.4–3.7)
No depressive symptoms	1 [Reference]	1 [Reference]	1 [Reference]
**Bullied online or at school in the past 12 months**			
Bullied	1.6 (1.5–1.8)	1.4 (1.2–1.7)	1.5 (1.3–1.8)
Not bullied	1 [Reference]	1 [Reference]	1 [Reference]
**In the past 7 days, how many days were you physically active for a total of at least 60 min**			
At least 5 days a week	1 [Reference]	1 [Reference]	— b
Less than 5 days a week	0.8 (0.8–0.9)	1.2 (1.0–1.5)	
**Grades**			
Mostly A’s and B’s	1 [Reference]	1 [Reference]	1 [Reference]
Mostly C’s, D’s, or F’s	1.9 (1.7–2.1)	2.2 (1.8–2.8)	2.4 (2.0–2.9)
**How often does one of your parents talk to you about school**			
About every day or once or twice a week	1 [Reference]	— b	— b
Once or twice a month or less	1.2 (1.1–1.3)		
**Do you agree or disagree that at least one of your teachers really cares and gives you support when you need it**			
Strongly agree or agree	1 [Reference]	1 [Reference]	1 [Reference]
Not sure or disagree or strongly disagree	1.4 (1.2–1.6)	1.6 (1.2–2.0)	1.7 (1.3–2.1)
**How often does your family give you love and support**			
Most of the time or all of the time	1 [Reference]	1 [Reference]	1 [Reference]
Little or no family love and support	1.3 (1.1–1.5)	1.5 (1.2–1.8)	1.4 (1.1–1.7)

a Adjusted for demographic characteristics (sex, sexual orientation, race, ethnicity, and grade level), school county, and all risk and protective factors.

b Removed from final model.

### Smoking

In bivariate analysis, all risk and protective factors were significantly associated with smoking in the past 30 days (Table 1), and these associations remained significant after adjusting for demographic characteristics and school county (Table 2).

In the full model, ORs were attenuated but remained significant for all risk and protective factors except talking to parents about school. This variable was removed from the final model (Table 3). The factors with the strongest associations with smoking included feeling like their parents think it is a little wrong or not wrong at all for them to smoke (aOR, 4.9), depressive symptoms in the past year (aOR, 2.7), housing instability (aOR, 2.4), and average grades of C or lower (aOR, 2.2).

### Vaping and smoking

In the bivariate analysis, all risk and protective factors were significantly associated with vaping and smoking in the past 30 days (Table 1), and these associations remained significant after adjusting for demographic characteristics and school county (Table 2).

In the full model, ORs were attenuated but remained significant for all risk and protective factors except physical activity and talking to parents about school; both variables were removed from the final model (Table 3). The factors with the strongest associations with vaping and smoking were feeling like their parents think it is a little wrong or not wrong at all for them to smoke (aOR, 4.6), depressive symptoms in the past year (aOR, 3.0), housing instability (aOR, 2.6), and average grades of C or lower (aOR, 2.4).

Overall, slight differences existed in the adjusted odds ratios for each risk factor by outcome. The risk factors with the consistently highest odds ratios for vaping, smoking, or doing both were if students felt their parent did not think it would be wrong if they smoked, if students reported being depressed in the past year, if students reported housing instability, and if average grades were a C or lower.

## Discussion

This study revealed several factors that increase the odds of vaping, smoking, or doing both among high school students in Maine. Factors with the greatest impact on a high school student’s odds of vaping, smoking, or doing both were the following: if a student feels that a parent thinks it is not wrong or slightly wrong for them to smoke, if a student reported having housing instability, if a student reported depressive symptoms in the past year, and if a student usually received grades of C or below. These findings are consistent with the findings of several other studies. The National Youth Tobacco Survey found a strong relationship between depression and smoking among adolescents, and students doing poorly in school were more likely than those not doing poorly to use a tobacco product (3). The 2016 Surgeon General’s Report also noted that students with lower academic achievement are more likely than those with higher academic achievement to begin tobacco use (14). A study that examined tobacco use among Somali middle and high school students in Minnesota found that parental antismoking norms protected against the use of all tobacco products (12). Our findings were similar: we found increased odds of vaping, smoking, or doing both when students felt their parents did not think it would be wrong for them to smoke. Our results were also similar to those of a study of college students in Texas, which found that depressive symptoms increased the likelihood of a vaping (9). Our finding of a relationship between poor school performance and use of tobacco products was similar to the findings of another study, which found an association between poor school performance and e-cigarette susceptibility among eighth graders (11).

Furthermore, our results showed that being bullied in the past year, perceiving smoking to have little to no health risk, feeling like a teacher does not care about them, and not feeling love and support from their family also significantly increased the odds of vaping, smoking, or doing both among high school students in Maine. The National Youth Tobacco Survey also indicated that lack of support from parents is associated with tobacco use among adolescents (3). Lastly, we found that the frequency of a student talking to their parents about school did not affect the odds of smoking or vaping and smoking. This finding differed from the findings of the National Youth Tobacco Survey, which indicated that lack of parental involvement was associated with use of tobacco products among students (3).

Unexpected results from our study were that fewer than 5 days per week of physical activity protected against vaping after adjustment for all variables. However, this was not true when we adjusted for demographic and school county variables only. This difference suggests a relationship between physical activity and one or more of the other risk and protective factors. However, decreased activity levels increased the odds of smoking. More research should be conducted to further examine whether physical activity is a risk factor or a protective factor for vaping and smoking among adolescents.

### Strengths and limitations

This study has several strengths. The MIYHS is a population-based survey, and data were weighted to be representative of the Maine high school-aged population. We had a diverse sample of participants from throughout the state. Additionally, the large sample size and overall response rate of 61.9% provided adequate statistical power for our analyses. This survey also included a wide variety of variables to assess multiple risk and protective factors. The variety enabled us to gain insight into factors that otherwise might have been overlooked, and it allowed for a comprehensive view of factors that increase the odds of a high school student vaping, smoking, or both.

This study also has several limitations. First, because only 2 of 4 survey versions asked about vaping, our assessment of risk and protective factors was limited. Second, because the survey was based on self-report among adolescents, the study was subject to recall bias and social desirability bias. To help mitigate some of the social desirability bias, the MIYHS survey is conducted anonymously and does not collect any personal identifiers. Third, students are asked only about the health risks posed by smoking, not vaping. The 2016 Surgeon General’s Report stated that low perceived harm of e-cigarettes was a commonly cited reason for use (14). If a question about the health risks of vaping had been included in the MIYHS, we might have seen a different response. Fourth, because the MIYHS is a cross-sectional survey, we could not assess cause and effect. Fifth, our study is generalizable only to the high school student population in Maine. Future studies must be conducted to further explore the relationships between various risk and protective factors in other states and regions.

### Public health implications

Understanding the potential risk and protective factors of an adolescent’s likelihood to vape, smoke, or both is critical for anyone in a high school student’s life, including parents, educators, physicians, and the students themselves. By understanding these factors, public health interventions can be appropriately tailored to reach the groups at risk of these behaviors. We have several recommendations for developing effective programs to decrease vaping and smoking rates among adolescents. We recommend that interventions include educating parents about smoking and vaping products, how these products work, nicotine addiction and its effect on the adolescent brain, and the factors that may increase or decrease the likelihood of their adolescent vaping, smoking, or doing both. Another recommendation is to increase services for adolescents to help with depression, bullying, and housing insecurity. Increasing availability and access to services, such as counseling, could help decrease the prevalence of some of these factors and potentially decrease the likelihood of an adolescent vaping or smoking. In addition, tobacco education should continue to be provided to adolescents in Maine and across the country. Finally, we recommend that research examine the association of risk and protective factors with different levels of use of e-cigarettes and cigarettes; for example, using the numbers of days of use during the past 30 days in analysis rather than just any use versus no use. 

### Conclusion

Several risk and protective factors, including a student’s perception of their parents’ view of them smoking, housing instability, and depressive symptoms, contribute to the likelihood of their vaping, smoking, or both. Although many of the risk and protective factors examined by this study were significant, some appear to have a greater effect than others and warrant heightened attention. Focus should be placed on educating parents about the risks of nicotine use among adolescents. Additionally, more services should be put in place to assist adolescents who experience depression, bullying, or housing instability. These additional services could potentially decrease vaping and smoking rates while providing these adolescents with the services they need.
